# Nuclear Imaging Study of the Pharmacodynamic Effects of Debio 1143, an Antagonist of Multiple Inhibitor of Apoptosis Proteins (IAPs), in a Triple-Negative Breast Cancer Model

**DOI:** 10.1155/2018/8494031

**Published:** 2018-12-02

**Authors:** Pierre-Simon Bellaye, Alexandra Oudot, Jean-Marc Vrigneaud, Olivier Raguin, Francis Bichat, Anne Vaslin, Hélène Maby-El Hajjami, Claudio Zanna, Grégoire Vuagniaux, Pierre Fumoleau, Franck Denat, François Brunotte, Bertrand Collin

**Affiliations:** ^1^Centre Georges-François Leclerc, Dijon, France; ^2^Oncodesign, Dijon, France; ^3^Debiopharm International SA, Lausanne, Switzerland; ^4^ICMUB, Dijon, France

## Abstract

**Background:**

Debio 1143, a potent orally available SMAC mimetic, targets inhibitors of apoptosis proteins (IAPs) members and is currently in clinical trials. In this study, nuclear imaging evaluated the effects of Debio 1143 on tumor cell death and metabolism in a triple-negative breast cancer (TNBC) cell line (MDA-MB-231)-based animal model.

**Methods:**

Apoptosis induced by Debio 1143 was assessed by FACS (caspase-3, annexin 5 (A5)), binding of ^99m^Tc-HYNIC-Annexin V, and a cell proliferation assay. ^99m^Tc-HYNIC-Annexin V SPECT and [^18^F]-FDG PET were also performed in mice xenografted with MDA-MB-231 cells.

**Results:**

Debio 1143 induced early apoptosis both *in vitro* and *in vivo* 6 h after treatment. Debio 1143 inhibited tumor growth, which was associated with a decreased tumor [^18^F]-FDG uptake when measured during treatment.

**Conclusions:**

This imaging study combining SPECT and PET showed the early proapoptotic effects of Debio 1143 resulting in a robust antitumor activity in a preclinical TNBC model. These imaging biomarkers represent valuable noninvasive tools for translational and clinical research in TNBC.

## 1. Background

The World Health Organization (WHO) reported that 1.7 million women were diagnosed with breast cancer in 2012 with a global number of 6.3 million women diagnosed with breast cancer between 2008 and 2012 [1]. Since the last WHO report in 2008, breast cancer incidence and mortality have increased by more than 20% and 14%, respectively. Breast cancer is also the leading cause of cancer-related death among women (522,000 deaths in 2012) and the most frequently diagnosed cancer in 140 of 184 countries worldwide [1]. The combination of surgery, radiation therapy, chemotherapy, and hormone therapy represents the common therapeutic strategies used nowadays in clinic to treat breast cancer. Clinical and pathologic features (based on conventional histology and immunohistochemistry) allow breast cancer classification as hormone-receptor positive (estrogen receptor (ER) and progesterone receptor (PR)), HER2 (human epidermal growth factor receptor 2) positive, and triple negative (ER, PR, and HER2 negative). This classification process is currently necessary for prognosis evaluation and individualized selection of therapy. Triple-negative breast cancer (TNBC) is a heterogeneous disease associated with a high risk of recurrence and poor prognosis. Therapeutic options for TNBC are currently limited to cytotoxic therapy, whereas other types of breast cancer expressing receptors are eligible for targeted therapies such as antihormonal or anti-HER2 therapies. Therefore, TNBC is considered as a real challenging disease since no targeted therapies has been approved yet. In this context, numerous new targets are currently under investigations for pharmacological purposes such as Notch signaling, Wnt/*β*-catenin, and Hedgehog pathways; EGFR, PARP1, mTOR, TGF-*β*, and angiogenesis inhibitors [2]. The targeting of the inhibitors of apoptosis proteins (IAPs), which are key negative regulators of programmed cell death, represents another promising approach in managing TNBC. Indeed, IAPs have been reported to be upregulated in most cancer types contributing to tumor cell survival and resistance to cancer therapy [3]. Among IAPs, four of them, namely, XIAP, cIAP1, cIAP2, and ML-IAP, negatively regulate apoptosis by downregulating the activity of caspases [4]. In addition to apoptosis, IAPs also influence a multitude of other cellular processes, such as ubiquitin-dependent signaling events that regulate activation of the nuclear factor *κ*B (NF*κ*B), which in turn drive the expression of genes important for inflammation, immunity, cell migration, and cell survival. It has been reported that XIAP protein expression was significantly correlated with a more aggressive tumor phenotype and decreased overall and disease-free survival, suggesting a prognostic value of XIAP for invasive ductal breast cancer with triple-negative phenotype [5]. IAPs are antagonized by the endogenous Second Mitochondria-derived Activator of Caspases (SMAC), also called DIABLO (Direct IAP-Binding Protein with Low PI). SMAC is released from mitochondria into the cytosol when mitochondria are damaged by apoptotic *stimuli* such as UV radiation [4]. Such a mechanism has paved the way for the design of SMAC-mimetic agents to promote apoptosis in cancer cells by antagonizing the activity of IAPs and create conditions in which apoptosis can proceed. A number of SMAC mimetics have been advanced into early clinical development for cancer treatment as single agent or in combination. Interestingly, it has been proposed that TNBC may be more sensitive to SMAC-mimetic drugs than other malignancies, suggesting that SMAC-mimetic could represent a targeted therapy of TNBC which remains to be discovered [4]. Recently, Debio 1143, a new potent orally available monovalent SMAC mimetic targeting multiple IAPs member, has been developed and is currently in clinical trials for cancer treatment [6]. Molecular imaging certainly represents a reliable technique to improve such a drug development since it is recognized to expedite cancer drug discovery, predict responders versus nonresponders to specific treatments, and help determine the overall effectiveness of therapies longitudinally [7]. In oncology, molecular imaging of glucidic metabolism with [^18^F]-FDG PET has already a crucial impact on several aspects from detection/staging to monitoring/predicting therapeutic effects in both preclinical and clinical settings, so that it remains a gold standard procedure in management of various malignancies. Nevertheless, even if [^18^F]-FDG uptake reflects the viable tumor cell fraction, it also accumulates in noncancer tissues (e.g., inflammatory lesions, brain, and heart) what can induce pitfalls in images interpretation. The combination of [^18^F]-FDG imaging with other modalities and/or probes able to image a specific biomarker related to the mechanism of action of the anticancer drugs to be tested is then a reliable way to circumvent these drawbacks. Most of anticancer drugs typically induce cell death through induction of apoptosis which can be noninvasively imaged with molecular imaging probe such as ^99m^Tc-HYNIC-Annexin V. Such a noninvasive imaging measure of apoptosis would therefore be helpful for demonstrating the efficacy of apoptosis-inducing treatments (e.g., Debio 1143) without requiring tissue sampling. As [^18^F]-FDG, ^99m^Tc-HYNIC-Annexin V is a well-known radiotracer and has been extensively assessed in preclinical and clinical settings, making it a safe and reliable probe in spite of a certain lack of specificity since it also labels necrosis [8]. In the current study, we combined SPECT and PET imaging techniques as pharmacodynamic biomarkers to measure the early proapoptotic and antitumor effects of Debio 1143 in a preclinical TNBC model. Using MDA*-*MB*-*231 xenografted mice, we successfully demonstrated that Debio 1143 induces apoptosis (^99m^Tc-HYNIC-Annexin V) at early time points and reduced glucidic metabolism ([^18^F]-FDG PET) over time, which was accompanied by a robust antitumor activity. These imaging biomarkers represent valuable noninvasive tools for translational research and might be useful for SMAC mimetic clinical development in TNBC.

## 2. Materials and Methods

Materials and methods are available in detail in Supplemental Methods.

### 2.1. Cell Culture (MDA-MB-231)

Breast adenocarcinoma MDA*-*MB*-*231 cells (European Collection of Authenticated Cell Cultures (ECACC), Salisbury, UK) have been cultured as a monolayer in RPMI 1640 containing 2 mM of L-glutamine (Lonza, Verviers, Belgium) supplemented with 10% fetal bovine serum (Lonza) at 37°C in a humidified atmosphere (5% CO_2_).

### 2.2. MTS Assay

MDA*-*MB*-*231 cells were plated in 190 *µ*L of medium per well in flat-bottom 96-well plates (Dutscher, Brumath, France). Plates were incubated in a drug-free culture medium at 37°C in a humidified atmosphere (5% CO_2_) for 24 hours before experiments. Then, cells have been incubated for 72 h with 10 increasing concentrations of Debio 1143 (5 pM to 10 *µ*M) and paclitaxel (0.5 pM to 1 *µ*M). Paclitaxel and Debio 1143 have been diluted in 0.3% DMSO. See details in Supplemental Methods.

### 2.3. Flow Cytometry

MDA*-*MB*-*231 cells were plated in 6-well flat-bottom plates (Dutscher) in 3.8 ml of RPMI 1640 and incubated at 37°C in a humidified atmosphere (5% CO_2_) for 24 hours before treatments. Debio 1143 (final concentration 0.3, 1, and 3 *µ*M in 0.3% DMSO) or staurosporine (final concentration 0.3, 1, and 3 *µ*M in 0.3% DMSO) was added to the corresponding wells, and control (vehicle) cells received 0.3% DMSO alone and incubated for 6 hours at 37°C in a humidified atmosphere (5% CO_2_). The effect of Debio 1143 and staurosporine on plasmatic membrane disruption was evaluated using an Annexin V-FITC/7-AAD KIT (Beckman Coulter, Roissy, France). Alternatively, the caspase-3 activity of MDA*-*MB*-*231 cells treated 24 h with Debio 1143 or staurosporine (both at a final concentration of 0.3, 1, and 3 *µ*M in 0.3% DMSO) was evaluated by FACS. Cells were plated in 25 cm^2^ flat-bottom flasks (Dutscher) in 9.5 ml of RPMI 1640 and incubated at 37°C under 5% CO_2_ for 24 hours before treatment. After incubation, cells were detached from the culture flask using trypsin, transferred to FACS tubes, and stained with PE Active Xaspase-3 Apoptosis KIT (BD Pharmigen, France). See details in Supplemental Methods.

### 2.4. ^99m^Tc-HYNIC-Annexin V

Annexin-V (A5) was functionalized with a bifunctional chelating agent (HYNIC) and was radiolabeled with technetium 99 m (99 mTc) according to an existing standardized protocol. Briefly, HYNIC-Annexin-V was provided by NIH and shipped frozen and stored at −80°C until use. Gamma-counting results are represented as the percentage of radioactivity bound to the apoptotic cells and will be determined according to %Bound = (A/A + B) × 100 (A: activity of the cell pellet; B: activity of the supernatant). See details in Supplemental Methods.

### 2.5. Animal Experiments

All animal experiments were performed according to the guidelines of the Ministère de la Recherche (Paris, France). All experiments were approved by the ethical committee of the “centre George François Leclerc” (Dijon, France). Tumors were induced subcutaneously by injecting 5.106 of MDA-MB-231 cells in 200 *µ*L of RPMI 1640 containing matrigel (50 : 50, v : v, BD Biosciences, France) into the right shoulder of female SCID mice.

*In vivo* evaluation of apoptosis was performed with SPECT-CT imaging (^99m^Tc-HYNIC-Annexin V). When tumors reached a mean volume of 340 mm^3^, ^99m^Tc-HYNIC-Annexin V SPECT-CT imaging was performed 6 and 24 hours after a single administration of vehicle (*p.o.*, *n*=8), Debio 1143 (*p.o.*, 100 mg/kg, *n*=8), or paclitaxel (IV, 7.5 mg/kg, *n*=8, Taxol®, 6 mg/mL, Bristol-Myers Squibb SpA, France). Mice were anesthetized through isoflurane inhalation for intravenous injection (tail vein) of 10–20 MBq of ^99m^Tc-HYNIC-Annexin V one hour prior the imaging study. At the end of the last image acquisition, the animals were sacrificed, and tumors were harvested and used for gamma counting in order to confirm image analyses.

*In vivo* evaluation of antitumor activity was performed with [^18^F]-FDG PET-CT. Treatments started when the tumors reached a mean volume of 100–200 mm^3^. The animals from group 1 (*n*=4) received daily *p.o.* administrations of vehicle for 14 consecutive days (D11 to D25), the animals from group 2 (*n*=4) received daily *p.o.* administrations of Debio 1143 at 100 mg/kg for 14 consecutive days (D11 to D25), and the animals from group 3 (*n*=4) received one IV injection of paclitaxel at 7.5 mg/kg every 7 days for a total of 2 injections (D18 and D25). [^18^F]-FDG-PET-CT imaging was performed in overnight fasted mice at one week of treatment (D18), two weeks of treatment (D25), and one week after last treatment (D32). Mice were anesthetized through isoflurane inhalation for intravenous injection (tail vein) of 15–20 MBq of [^18^F]-FDG 30 minutes prior the imaging study. Alternatively, mice receiving vehicle, Debio 1143, or paclitaxel received an intravenous injection (tail vein) of 15–20 MBq of [^18^F]-FDG and were immediately imaged by dynamic PET-CT for 240 seconds to evaluate tracer circulation and tumor perfusion.

At the end of the last imaging, the mice were intraperitoneally injected with an overdose of pentobarbital for euthanasia and tumors harvested for gamma counting (Perkin Elmer, France). See details in Supplemental Methods.

### 2.6. Statistical Analysis

All results are presented as mean ± SEM. Statistical analysis was determined using one-way (^99m^Tc-HYNIC-Annexin V experiments) or two-way ANOVA ([^18^F]-FDG PET-CT). Analysis was performed with GraphPad Prism 6.0 (GraphPad Software Inc.), and in all cases, a *p* value less than 0.05 was considered significant.

## 3. Results

### 3.1. The Cytotoxic Activity of Debio 1143 on Human Breast Adenocarcinoma Cells Is Comparable to Paclitaxel

**Figure 1:**
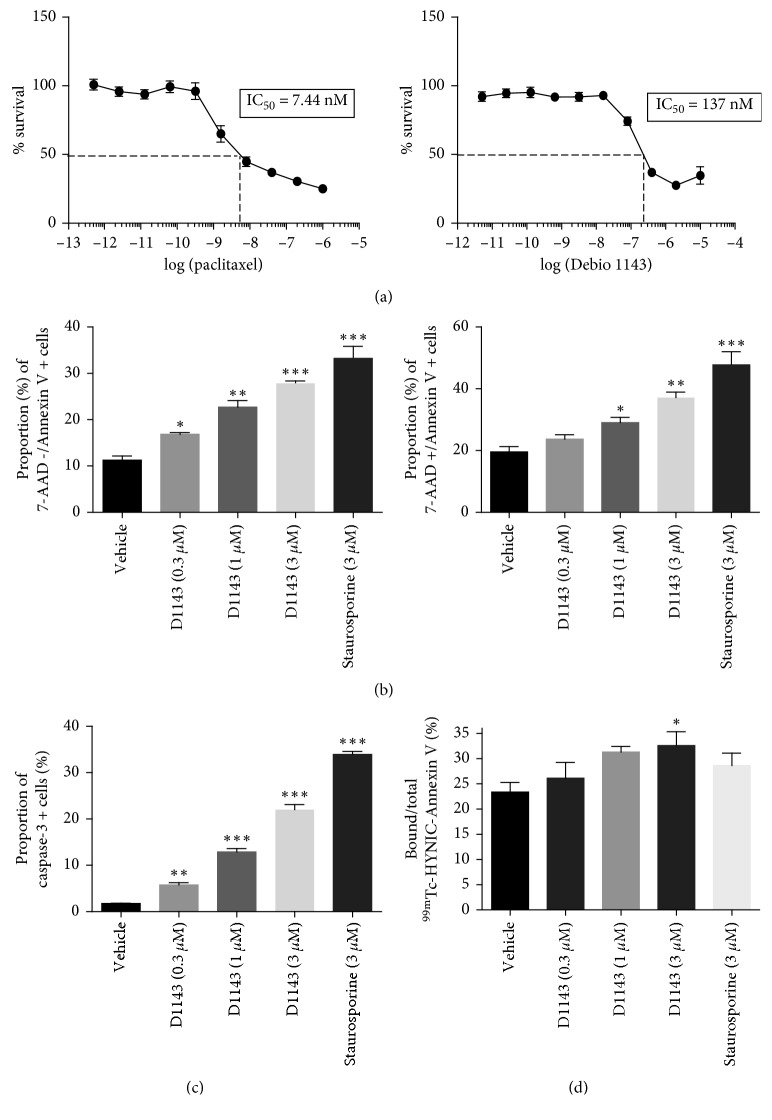
D1143 induces apoptosis of human breast adenocarcinoma cells. (a) Viable *MDA-MB-231* cells (%) after treatment with increasing concentration of paclitaxel (left panel) or D1143 (right panel) for 72 h. Paclitaxel and D1143 are expressed as log[concentration] for IC50 determination. Results are presented as mean ± SEM; *n*=8. (b) Annexin-V+/7-AAD- (left panel) and Annexin-V+/7-AAD+ (right panel) *MDA-MB-231* cells (%) after treatment with D1143 (0.3 *µ*M, 1 *µ*M, and 3 *µ*M) or staurosporine (3 *µ*M) for 6 h. Results are presented as mean ± SEM; *n*=4; ^*∗*^ *p* < 0.05, ^*∗∗*^ *p* < 0.01, ^*∗∗∗*^ *p* < 0.001. (c) Active caspase-3 positive *MDA-MB-231* cells (%) after treatment with D1143 (0.3 *µ*M, 1 *µ*M, and 3 *µ*M) or staurosporine (3 *µ*M) for 6 h. Results are presented as mean ± SEM; *n*=4; ^*∗∗*^ *p* < 0.01, ^*∗∗∗*^ *p* < 0.001. (d) Bound/Total ^99m^Tc-HYNIC-Annexin V *MDA-MB-231* cells (%) after treatment with D1143 (0.3 *µ*M, 1 *µ*M, and 3 *µ*M) or staurosporine (3 *µ*M) for 6h. Results are presented as mean ± SEM; *n*=4; ^*∗*^ *p* < 0.05.

The incubation of MDA-MB-231 cells with increasing concentration of Debio 1143 and paclitaxel demonstrated a dose-dependent cytotoxic activity of both drugs on human breast adenocarcinoma cells. The mean IC50 of D1143 was 137 nM, while the mean IC50 of paclitaxel was 7.44 nM (Figure 1). Our results confirm the findings of previous studies which report an IC50 of 144 nM for Debio 1143 [9].

### 3.2. Debio 1143 Induces Apoptosis of Human Breast Adenocarcinoma Cells

After 6 hours of incubation of MDA-MB-231 cells with Debio 1143, a significant dose-dependent increase of cells in early apoptosis (Annexin-V+/7-AAD-) was observed compared to vehicle-treated cells (Figure 1). This increase in early apoptosis was observed starting at 0.3 *µ*M with a maximal effect at 3 *µ*M of Debio 1143. Staurosporine, used as positive control in this experiment, also induced a significant increase in early apoptosis in MDA-MB-231 cells (Figure 1). Interestingly, Debio 1143 also induced a significant increase in late apoptosis/necrosis (Annexin-V+/7-AAD+) of MDA-MB-231 cells starting at 1 *µ*M and increased with dose (Figure 1). These results were confirmed by a dose-dependent increase in proportion of cells harboring active caspase-3, the major effector of apoptosis, after Debio 1143 treatment (Figure 1). Furthermore, gamma counting of MDA-MB-231 cells after staining with ^99m^Tc-HYNIC-Annexin V, which specifically stains Annexin-V positive cells, demonstrated that Debio 1143 (3 *µ*M) induced an increase in cells presenting Annexin-V (Figure 1). All together, these results highlight the proapoptotic effects of Debio 1143 on human breast adenocarcinoma cells.

### 3.3. Debio 1143 Induces Tumor-Apoptosis In Vivo in a Human Breast Adenocarcinoma Murine Model

**Figure 2:**
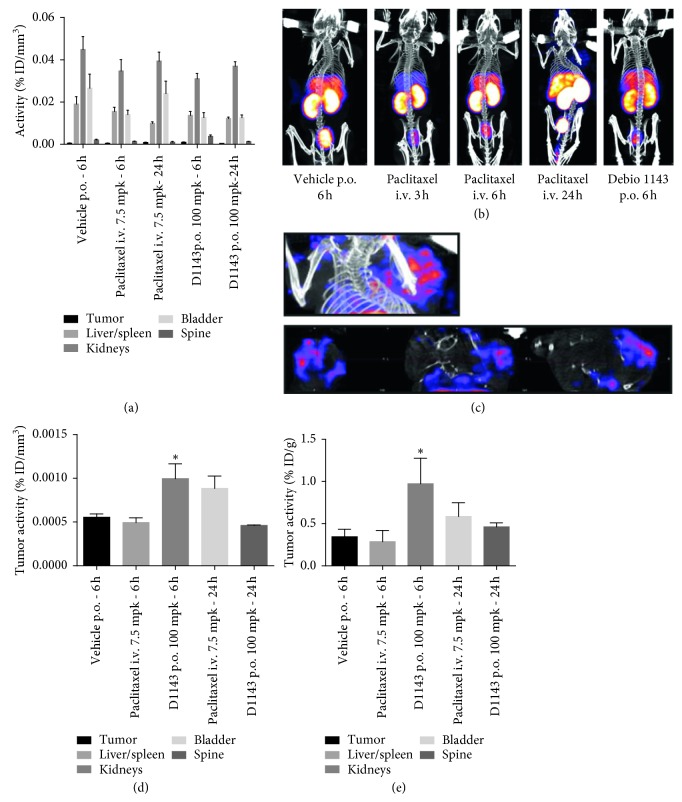
D1143 induces tumor apoptosis *in vivo* in a human breast adenocarcinoma murine model. (a) *In vivo* biodistribution of ^99m^Tc-HYNIC-Annexin V in tumor (*MDA-MB-231* cells) bearing SCID mice (tumor in the right shoulder) 6 h and 24 h after receiving paclitaxel (iv), D1143 (po), or vehicle as control. Liver/spleen, kidneys, bladder, spine, and tumor activity are expressed as % ID/mm^3^. Results are presented as mean ± SEM; *n*=8. (b) Representative SPECT pictures of ^99m^Tc-HYNIC-Annexin V in tumor (*MDA-MB-231* cells) bearing SCID mice 6 h and 24 h after receiving paclitaxel (iv), D1143 (po), or vehicle as control. (c) Representative tumor-centered SPECT pictures of tumor (*MDA-MB-231* cells) bearing SCID mice 6 h after receiving D1143 (po). (d) Specific ^99m^Tc-HYNIC-Annexin V tumor activity (% ID/mm^3^) of tumor- (*MDA-MB-231* cells-) bearing SCID mice (tumor in the right shoulder) 6 h and 24 h after receiving paclitaxel (iv), D1143 (po), or vehicle as control. Results are presented as mean ± SEM; *n*=8; ^*∗*^ *p* < 0.05. (e) Gamma counting of ^99m^Tc-HYNIC-Annexin V in tumors in SCID mice 6h and 24h after receiving paclitaxel (iv), D1143 (po), or vehicle as control (%ID/g). Results are presented as mean ± SEM; *n*=8 and *n*=2 for the paclitaxel group 6 h, ^*∗*^ *p* < 0.05.

^99m^Tc-HYNIC-Annexin V SPECT-CT imaging experiments were carried out when tumors reached a mean volume of 340 mm^3^. Imaging was performed at 6 h after treatment for vehicle-treated mice and at 6 and 24 h after treatment for paclitaxel- and Debio 1143-treated mice. One hour after ^99m^Tc-HYNIC-Annexin V administration, mice from all group showed an apparent similar whole body distribution of radioactivity localized mainly in kidneys, bladder, and liver concentrating more than 80% of overall radioactive signal as previously described in the literature ([8]; Figures 2 and 2). A weak ^99m^Tc-HYNIC-Annexin V signal was observed in tumors from vehicle-treated mice, comparable with signal observed in paclitaxel-treated mice. Interestingly, a significant increase in tumor ^99m^Tc-HYNIC-Annexin V signal was observed at 6h following Debio 1143 treatment (Figures 2 and 2). An increase in ^99m^Tc-HYNIC-Annexin V signal was also observed after 24 h of paclitaxel although not significant (Figure 2). These results were consistent with *ex vivo* gamma counting of tumors with an increase of ^99m^Tc-HYNIC-Annexin V tumor uptake 6 h after Debio 1143 compared to vehicle-treated mice (Figure 2). All together, these results demonstrate that Debio 1143 specifically induces tumor apoptosis *in vivo* in a human breast adenocarcinoma murine model.

### 3.4. *In Vivo* Evaluation of the Antitumor Activity of Debio 1143 by [^18^F]-FDG PET-CT

**Figure 3:**
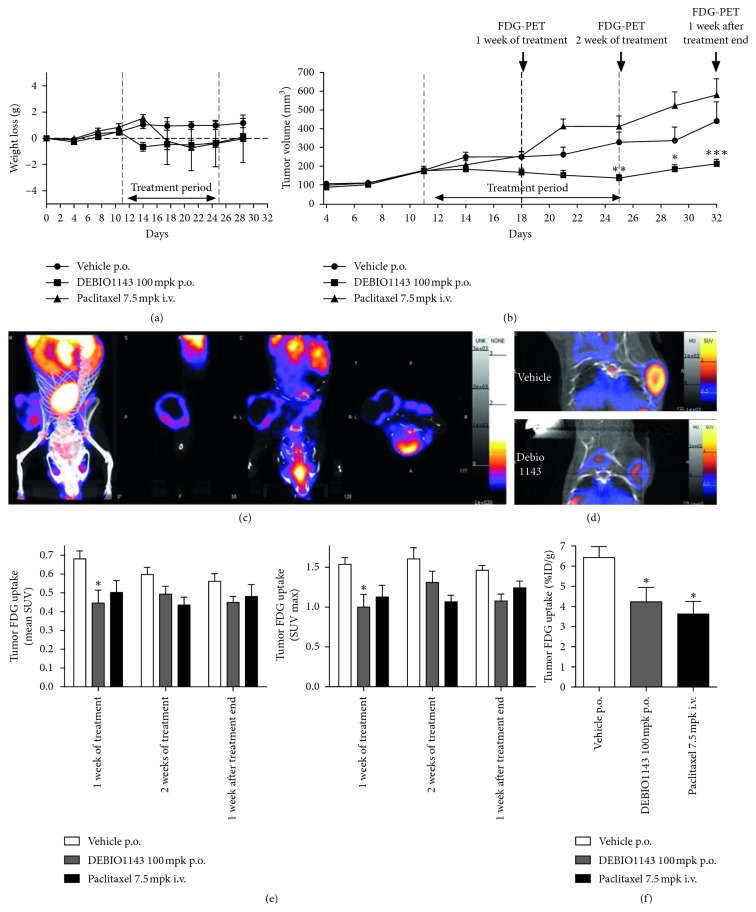
*In vivo* evaluation of the antitumor activity of D1143 by [18F]-FDG PET-CT. (a) Weight loss (g) monitoring of tumor-bearing SCID mice receiving D1143, paclitaxel, or vehicle as control. Treatment started at D11 and ended at D25. Mice were sacrificed at D32. Results are presented as mean ± SEM; *n*=8. (b) Tumor volume (mm^3^) of tumor-bearing SCID mice receiving D1143, paclitaxel, or vehicle as control. Treatment started at D11 and ended at D25. Results are presented as mean ± SEM; *n*=8; ^*∗∗*^ *p* < 0.01, ^*∗∗∗*^ *p* < 0.01. (c) Representative [^18^F]-FDG PET-CT picture of tumor-bearing SCID mice (tumor in the right shoulder) receiving vehicle at D32. (d) Representative [^18^F]-FDG PET-CT picture of tumor-bearing SCID mice (tumor in the right shoulder) receiving vehicle or D1143 at D32. (e) Tumor [^18^F]-FDG uptake in SCID mice receiving vehicle, D1143, or paclitaxel. Measures have been performed at D18 (1 week of treatment), D25 (2 weeks of treatment), and D32 (1 week after treatment). Results are expressed as mean SUV (left panel) and SUV max (right panel). Results are presented as mean ± SEM; *n*=8, ^*∗*^ *p* < 0.05. (f) Gamma counting of [^18^F]-FDG in tumors of SCID mice receiving paclitaxel (iv), D1143 (po), or vehicle as control (%ID/g). Results are presented as mean ± SEM; *n*=8; ^*∗*^ *p* < 0.05.

**Figure 4:**
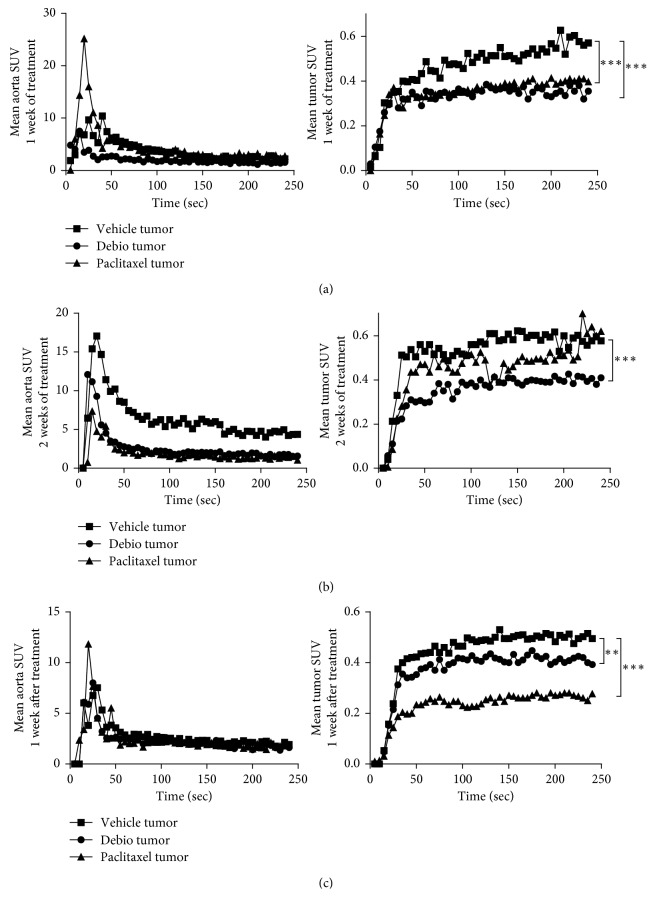
*In vivo* dynamic [18F]-FDG PET-CT. (a) Dynamic [18F]-FDG PET-CT performed on tumor-bearing SCID mice receiving paclitaxel, D1143, or vehicle at D18 (1 week of treatment). Mean aorta SUV (left panel) and mean tumor SUV (right panel) have been measured every 5 seconds for 240 seconds. (b) Dynamic [18F]-FDG PET-CT performed on tumor-bearing SCID mice receiving paclitaxel, D1143, or vehicle at D25 (week of treatment). Mean aorta SUV (left panel) and mean tumor SUV (right panel) have been measured every 5 seconds for 240 seconds. (c) Dynamic [18F]-FDG PET-CT performed on tumor-bearing SCID mice receiving paclitaxel, D1143, or vehicle at D32 (1 week after treatment). Mean aorta SUV (left panel) and mean tumor SUV (right panel) have been measured every 5 seconds for 240 seconds.

After tumor induction, mice received vehicle, Debio 1143, or paclitaxel for 2 weeks. Treatment started when mean tumor volume reached approximately 120–170 mm^3^ (D11). Mice received corresponding treatment from D11 to D25 (2 weeks) and were left untreated for another week up to D32. While mice receiving vehicle continued to gain weight throughout the experiment, paclitaxel and Debio 1143 induced a slight and transient decrease of body weight recovered once treatments ended (Figure 3). Tumor volume increased regularly and similarly in vehicle-treated mice from D11 (treatment initiation) to D32 (end of experiment; Figure 3). Paclitaxel did not induce any decrease in tumor growth throughout the experiment, while Debio 1143 displayed a significant antitumor activity after 2 weeks of treatment (D25) that was sustained up to D32 (Figure 3). [^18^F]-FDG PET-CT was performed on D18 (1 week of treatment), D25 (2 weeks of treatment), and D32 (1 week after treatment end). [^18^F]-FDG uptake measured by SUV (standardized uptake values) max and mean SUV was significantly lower in Debio 1143-treated mice compared to vehicle at D18 (Figures 3–3). [^18^F]-FDG uptake remained lower in Debio 1143-treated mice compared to vehicle throughout the experiment but not significantly at D25 and D32 (Figures 3–3). Paclitaxel also reduced not significantly [^18^F]-FDG uptake as compared to vehicle-treated mice (Figures 3–3). Interestingly, gamma counting performed on tumors at D32 (1 week after treatment end) confirmed our imaging results with a significant lower tumor [^18^F]-FDG uptake in Debio 1143 and paclitaxel-treated mice compared to vehicle (Figure 3). We also performed dynamic [^18^F]-FDG PET-CT imaging for 4 minutes after injection on all groups at D18, D25, and D32 to evaluate tumor perfusion. Interestingly, dynamic monitoring of mean tumor SUV (every 5 seconds for 240 seconds) showed a significant decrease in tumor perfusion in mice treated with Debio 1143 and paclitaxel at D18 and D32 and only in mice treated with D1143 at D25 (Figures 4(a)–4(c)). No changes were observed in mean aorta SUV (control [^18^F]-FDG SUV). All together, these results demonstrate the antitumor activity of Debio 1143 and highlight [^18^F]-FDG PET-CT imaging as a reliable method to follow the activity of Debio 1143 in human breast adenocarcinoma tumors in a noninvasive manner.

## 4. Discussion

In order to improve the management of malignancies, it is now well established that an early and reliable assessment of therapy response is a crucial issue. It allows guidance of the oncologist to the best options for the patients: modulations of the doses, treatment switching, or treatment combinations. In the current study, using two different molecular imaging modalities (SPECT-CT and PET-CT), we assessed the effect of Debio 1143, a new potent oral SMAC mimetic, as a single agent in a preclinical model of TNBC, in immunodeficient mice xenografted with MDA-MB-231 cells. The xenografted models still constitute a major preclinical screen for the development of novel cancer therapeutics, included human-targeted therapies. Despite limitations, these models have identified clinically efficacious agents, suggesting that they are still a “workhorse” of the pharmaceutical industry [10]. TNBC represents 15–20% of breast cancers and remains a challenging disease regarding its aggressive nature, its poor prognosis, and the lack of targeted therapies. As no well-defined molecular targets have been described so far, cytotoxic chemotherapy is currently the only treatment option for TNBC whose major drawback is an unacceptable deterioration in the quality of life. Currently, paclitaxel is commonly used in clinical practice to treat TNBC. However, the clinical efficacy of paclitaxel has been weakened by the development of drug resistance and the emergence of side-effects, including neutropenia and neurotoxicity [11]. Paclitaxel induces apoptosis by targeting microtubules and resulting in cell cycle arrest [12]. Although paclitaxel has been shown to eliminate most tumor cells including TNBC, paclitaxel resistance has been estimated to cause treatment failure in more than 90% of patients [13]. Therefore, the development of alternative therapeutic strategies is essential. Inhibitor of apoptosis proteins (IAPs) play key roles in resistance to cell death induced by a variety of anticancer drugs in various indications including in TNBC, and thus are promising drug targets [4]. Debio 1143 (a.k.a. AT-406 or SM-406) is a monovalent, orally available, small molecule antagonist of IAPs in clinical development that has demonstrated potent single-agent antitumor activity in multiple models of human cancer such as lung adenocarcinoma [14, 15], head and neck squamous cell carcinoma [16], and TNBC [9, 17]. Debio 1143 has also been shown to work synergistically with conventional chemotherapeutic agents (such as taxanes) or radiotherapy RT in nonclinical cancer models [14, 16]. SMAC mimetics have been shown to promote apoptosis by inhibiting IAP-mediated caspase repression [18]. *In vitro* SMAC-mimetics treatment has been shown to increase Annexin-V positive cells and activate caspases-3 and -8 in various cancer cell lines [16, 19, 20]. Our results are in line with previous studies and confirm the increase in Annexin-V and activation of caspase-3 after Debio 1143 treatment in MDA-MB-231 cells. Our results also demonstrate that, this increase in Annexin-V can be measured in tumor *in vivo* in a preclinical model of breast adenocarcinoma with radiolabelled ^99m^Tc-HYNIC-Annexin V. This tool could represent a reliable way to monitor early apoptosis induced by anticancer agents in order to evaluate early treatment efficacy and allow improvement of therapeutic strategies.

Interestingly, Debio 1143 presented a higher antitumor activity *in vivo* in comparison with paclitaxel despite an apparent higher intrinsic cytotoxic activity of paclitaxel *in vitro* suggesting that targeting IAPs may offer the potential for a greater therapeutic window than conventional chemotherapy *in vivo*. In addition, our results show that Debio 1143 and placlitaxel presented differentiated proapoptotic effects over time *in vivo*. Debio 1143 induced an earlier and stronger cancer cell apoptosis as early as 6 h after treatment, whereas paclitaxel induced-apoptosis was only detectable (although not significant) 24 h after treatment. Apoptosis is an early event expected to occur after successful chemotherapy and is highly predictive of treatment success. Thus, apoptosis quantification represents a major way to assess therapy response. Annexin V (A5) has been widely used in basic and clinical research as an apoptosis marker in conjunction with propidium iodide to distinguish between apoptotic and necrotic cells. Therefore, it has been labeled with radionuclides for measuring apoptosis *in vitro* and *in vivo* in animal models and patients [8, 21, 22]. ^99m^Tc-HYNIC-Annexin V, used in the current study, is the most widely applied probe in preclinical and clinical settings for A5 imaging [23]. Kemerink et al. demonstrated that highest uptake of ^99m^Tc-HYNIC-Annexin V in humans was observed in the kidneys followed by the liver and spleen [24]. These results are in accordance with our findings in mice where the highest uptake was found in the kidney at 6h and 24h.

Moreover, ^99m^Tc-HYNIC-Annexin V showed a fast blood clearance with more than 90% of the tracer cleared with a half-life of 24 min [24], allowing imaging at 6 h after injection. Therefore, ^99m^Tc-HYNIC-Annexin V has been used successfully to assess therapy response in patients after radiation therapy or chemotherapy [25–27]. ^99m^Tc-HYNIC-Annexin V uptake has also been demonstrated to predict prognostic value and efficacy of anticancer therapies. In our study, Debio 1143 induced a significantly higher tumor (MDA-MB-231) uptake of ^99m^Tc-HYNIC-Annexin V compared to vehicle and paclitaxel. In parallel, Debio 1143 showed an improved efficacy in preventing tumor growth compared to vehicle and paclitaxel after 1 and 2 weeks of treatment and remained 1 week after treatment arrest confirming the predictive value of ^99m^Tc-HYNIC-Annexin V tumor uptake on therapy efficacy. Unexpectedly, paclitaxel did not induce a strong *in vivo* apoptosis in our study and, in parallel, did not prevent tumor growth. Despite some controversy, MDA-MB-231 has been demonstrated to be rather insensitive to paclitaxel compared to other TNBC cells [28–30]. Interestingly, Panayotopoulou et al. identified, by high throughput screening, that SMAC mimetics were able to eliminate MDA-MB-231 short-term paclitaxel resistance suggesting a benefit of such drugs for TNBC patients [31]. Similar results have also been found in other cancer types including ovarian cancer [32], non-small cell lung cancer [14], and breast cancer [33], in which SMAC mimetics were able to potentiate the effect of standard chemotherapy, including paclitaxel [14, 34, 35]. However, this study did not evaluate the efficacy of the combination of SMAC mimetics and paclitaxel with [^18^F]-FDG PET imaging. Moreover, Panayotopoulou et al. identified that long-term paclitaxel was associated with desensitization to SMAC mimetics. Therefore, combination therapy of SMAC mimetics and short-term paclitaxel could be an effective therapeutic strategy for TNBC.

Most interestingly, the effect of the SMAC mimetic birinapant on caspase-3 activation has recently been investigated by *in vivo* imaging [36]. In this study, Yang et al. used a specific caspase-3 PET radiotracer, [^18^F]ICMT-11, and demonstrated that birinapant induced *in vitro* a rapid and transient activation of caspase-3 on MDA-MB-231 cells 6 h after treatment. Moreover, a similar activation of caspase-3 was also shown *in vivo* in a preclinical model of colon cancer. These results are in accordance with Debio 1143 presented in the current study using ^99m^Tc-HYNIC-Annexin V. In addition, Yang et al. also observed a decrease [^18^F]-FDG uptake and a delay in tumor growth *in vivo* after birinapant treatment similarly to what was observed with Debio 1143 in our study. Interestingly, the *in vivo* activation of caspase-3 and decrease in [^18^F]-FDG uptake induced by birinapant was only transient and returned to baseline 24 h and 48 h after treatment highlighting the need of multiple dosing of SMAC mimetics to elicit antitumor activity as monotherapy.

## 5. Conclusions

[^18^F]-FDG PET is nowadays the main tool for detection, staging, and monitoring of tumor clinically. However, [^18^F]-FDG uptake accumulates in noncancer tissues and can be influenced by physiologic uptake of FDG (for example, infection and inflammation) [37].

Moreover, some adenocarcinoma are characterized by low-grade or absence of FDG uptake [38, 39]. In our study, we demonstrate that both ^99m^Tc-HYNIC-Annexin V and [^18^F]-FDG PET data can be associated to predict therapy efficacy and outcome. Therefore, the combination of [^18^F]-FDG PET and ^99m^Tc-HYNIC-Annexin V appears as a reliable and noninvasive way to monitor early therapy efficacy and subsequent tumor activity in TNBC patients.

## Data Availability

The datasets used and/or analyzed during the current study are available from the corresponding author on reasonable request.

## Abbreviations

7-AAD: 7-Aminoactinomycin D
A5: Annexin-5
DIABLO: Direct IAP-Binding Protein with Low PI
DMSO: Dimethylsulfoxide
EGFR: Epidermal growth factor receptor
ER: Estrogen receptor
FDG: Fluorodeoxyglucose
HER2: Human epidermal growth factor receptor 2
IAP: Inhibitor of apoptosis proteins
IV: Intravenous
NIH: National Institute
PET: Positron emission tomography
PR: Progesterone receptor
SMAC: Second Mitochondria-derived Activator of Caspases
SPECT: Single-photon emission tomography
SUV: Standardized uptake values
TGF-*β*: Transforming growth factor-*β*
TNBC: Triple-negative breast cancer
WHO: World Health Organization.
