# Supplementary material for: Nuclear Imaging Study of the Pharmacodynamic Effects of Debio 1143, an Antagonist of Multiple Inhibitor of Apoptosis Proteins (IAPs), in a Triple-Negative Breast Cancer Model
Source: Contrast Media Mol Imaging. 2018 Dec 2;2018:8494031. doi: 10.1155/2018/8494031 (PMC6305031; doi:10.1155/2018/8494031)
Supplement: Supplementary Materials — Materials and methods are provided in detail in the Supplementary Materials file. [file 8494031.f1.pdf]

Supplemental Material and methods

Cell culture (MDA-MB-231)

Breast adenocarcinoma MDA-MB-231 cells (European Collection of Authenticated Cell Cultures (ECACC), Salisbury, UK) have been cultured as a monolayer in RPMI 1640 containing 2mM of L-glutamine (Lonza, Verviers, Belgium) supplemented with 10% fetal bovine serum (Lonza) at 37°C in a humidified atmosphere (5% CO_2).

MTS assay

MDA-MB-231 cells were plated in 190 µL of medium per well in flat bottom 96-well plates (Dutscher, Brumath, France). Plates were incubated in drug free culture medium at 37°C in a humidified atmosphere (5% CO2) for 24 hours before experiments. Then, cells have been incubated for 72h with 10 increasing concentrations of DEBIO 1143 (5 pM to 10µM) and Paclitaxel (0.5 pM to 1 µM). Paclitaxel (Taxol®, 6 mg/mL, Bristol-Myers Squibb SpA, France) and DEBIO 1143 have been diluted in 0.3% DMSO. The *in vitro* cytotoxic activity of DEBIO 1143 and Paclitaxel was revealed at 72h by addition of 40 µL of a 0.22 µm filtered freshly combined solution of MTS (3-(4,5-dimethylthiazol-2-yl)-5-(3-carboxymethoxy phenyl)-2-(4-sulfophenyl)-2H-tetrazolium (Promega, Charbonnières, France) and PMS (phenazine methosulfate, Sigma) in DPBS (Lonza). Optical density (OD) was measured at 490 nm in each well using VICTOR3™ 1420 multilabeled counter (PerkinElmer, Courtaboeuf, France). Optical density values were corrected by background (blank) and corresponded to the mean of 4 experimental measurements. Dose-response curves were plotted using Graphpad (GraphPad Software, CA, USA). The IC_{50} drug concentration to obtain a 50% inhibition of cell proliferation values was determined from semi-log curves.

Flow cytometry

MDA-MB-231 cells were plated in 6-well flat-bottom plates (Dutscher) in 3.8 ml of RPMI 1640 and incubated at 37°C in a humidified atmosphere (5% CO2) for 24 hours before treatments. DEBIO 1143 (final concentration 0.3, 1 and 3 µM in 0.3%DMSO) or Staurosporine (final concentration 0.3, 1 and 3 µM in 0.3%DMSO) were added to the corresponding wells, (Control (vehicle) cells received 0.3% DMSO alone), *and* incubated for 6 hours at 37°C in a humidified atmosphere (5% CO2). The effect of DEBIO 1143 and Staurosporine on plasmatic membrane disruption was evaluated using an Annexin V-FITC/7-AAD KIT (Beckman Coulter, Roissy CDG, France). Briefly, cells were washed and incubated in ice-cold binding buffer. Then, 10 µL of Annexin-V FITC solution and 20 µL of 7-AAD viability dye were added to 100 µL of

cells suspension and incubated 15 minutes on ice in the dark. After incubation, cells were analyzed by FACS within 30-60 minutes. Staining cells were analyzed with a CyFlow® space flow cytometer (Partec S.A.R.L., Sainte Geneviève des Bois, France) using a 488 nm wavelength laser excitation. The acquisition was stopped after a total of 10,000 FSC/SSC-gated cells were collected for each sample. Percentages of viable cells (7-AAD$^-$ / Annexin-5$^-$), in early apoptosis (7-AAD$^-$ / Annexin-5$^+$) and in late apoptosis or necrosis (7-AAD$^+$) were determined. Alternatively, the caspase-3 activity of *MDA-MB-231* cells treated 24h with Debio 1143 or Staurosporine (both at a final concentration of 0.3, 1 and 3 µM in 0.3%DMSO) was evaluated by FACS. Cells were plated in 25 cm^2 flat-bottom flasks (Dutscher) in 9.5 ml of RPMI 1640 and incubated at 37°C under 5% CO2 for 24 hours before treatment. After incubation, cells were detached from the culture flask using trypsin, transferred to FACS tubes and stained with PE Active Xaspase-3 Apoptosis KIT (BD Pharmigen, France). Briefly, one million (1.10^6) cells were fixed and permeabilized in ice-cold BD Cytofix/CytopermTM buffer for 20 minutes. Then, cells were pelleted and washed with BD Perm/WashTM buffer. Cells were then incubated at room temperature for 30 minutes in the BD Perm/WashTM buffer in the presence of the PE conjugated antibody directed against activated Caspase 3. Stained cells were analyzed with a CyFlow® space flow cytometer (Partec S.A.R.L., Sainte Geneviève des Bois, France) using a 488 nm wavelength laser excitation. The acquisition was stopped after a total of 10,000 FSC/SSC-gated cells are collected for each sample.

^{99m}Tc-HYNIC-Annexin V

Annexin-V (A5) was functionalized with a bifunctional chelating agent (Hynic) and was radiolabeled with technetium ^{99}m (^{99m}Tc) according to an existing standardized protocol. Briefly, Hynic-Annexin-V (Hynic-A5) was provided by NIH and shipped frozen and stored at –80°C until use. Thirty to fifty mCi (0.5 ml) of ^{99m}Tc pertechnetate were added to Hynic-A5 and 0.3 mL of stannous tricine saline solution. After a 15-min incubation at room temperature, quality control was performed to validate that ^{99m}Tc-HYNIC-Annexin V was obtained with the suitable radiolabeling yield (*i.e* > 90%). Quantification was obtained with ITLC-SG (Agilent, France) strips developed in ACD buffer and read with an AR-2000 planar imager (Bioscan, USA). After incubation with ^{99m}Tc-HYNIC-Annexin V, cells were detached from the well using trypsin, and were separated from supernatant by centrifugation (5 min – 500 g) and washed with cold PBS and centrifuged (5 min – 500 g) for ^{99m}Tc-HYNIC-Annexin V radiolabeling. ^{99m}Tc-HYNIC-Annexin V cell radiolabeling (target specific activity about 6 MBq / µg) was performed to obtain final ^{99m}Tc-HYNIC-Annexin V concentrations of 2.5 µg /

mL. Briefly, 10 µL of ^{99m}Tc-HYNIC-Annexin V (up to 2.1 MBq) were added to 100 µL of cell suspension, completed with 20 µL of PBS (final volume = 130 µL). After gentle swirl, the mix was be incubated at room temperature for 30 minutes. The radiolabeled cells were then washed with 500 µL of cold PBS and pelleted again by centrifugation (5 min – 500 g). Supernatant and cell pellets were separated for gamma-counting (Perkin Elmer, France). Gamma-counting results is represented as the percentage of radioactivity bound to the apoptotic cells and will be determined according to: $\%_{Bound}$ = (A / A+B) x 100 (A: activity of the cell pellet; B: activity of the supernatant).

Animal experiments

All animal experiments were performed according to the guidelines of the Ministère de la Recherche (Paris, France). All experiments were approved by the ethical committee of the "centre George François Leclerc" (Dijon, France). Tumors were induced subcutaneously by injecting 5.10^6 of MDA-MB-231 cells in 200 µL of RPMI 1640 containing matrigel (50:50, v:v, BD Biosciences, France) into the right shoulder of female SCID mice. MDA-MB-231 tumor cell implantation was performed 24 to 72 hours after a whole body irradiation with a γ-source (1.44 Gy, ^{60}Co, BioMep, France). The day of tumor induction was considered as the day 0 (D0). Body weights were monitored twice a week. The length and width of the tumors were measured and recorded twice a week with calipers and the tumor volumes were estimated by the formula: tumor volume = 0.5 x length x width^2.

In vivo evaluation of apoptosis was performed with SPECT-CT imaging (^{99m}Tc-HYNIC-Annexin V). When tumors reached a mean volume of 340 mm^3, ^{99m}Tc-HYNIC-Annexin V SPECT-CT imaging was performed 6 hours and 24 hours after a single administration of vehicle (*p.o*, n = 8), Debio 1143 (*p.o*, 100 mg/kg, n = 8), or paclitaxel (IV, 7.5 mg/kg, n = 8). Mice were anesthetized through isoflurane inhalation for intravenous injection (tail vein) of 10-20 MBq of ^{99m}Tc-HYNIC-Annexin V one hour prior the imaging study. CT acquisitions (55kVp, 34 mAs) were first acquired during 10-15 min, followed by helical SPECT acquisitions (NanoSPECT^{TM}/CT, Bioscan, USA) with 90-120 second per projection frame. At the end of the last image acquisition, the animals are sacrificed, and tumors harvested and used for gamma counting in order to confirm image analyses. The SPECT/CT fusion images were obtained using the InVivoScope^{TM} software (Bioscan, USA). Each scan was then visually interpreted and 3D regions of interest corresponding to the tumor and whole body were manually drawn in order to determine their radioactivity content. *In vivo* quantification was obtained by accurate calibration of the NanoSPECT^{TM}/CT. Radioactivity contents from image analysis are expressed

in Bq, converted to percentage of injected dose (% ID) and compared to those determined by *ex vivo* gamma counting (Perkin Elmer, France).

In vivo evaluation of antitumor activity was performed with [^{18}F]-FDG PET-CT. Treatments started when the tumors reached a mean volume of 100-200 mm^3. The animals from group 1 (n = 4) received daily *p.o* administrations of vehicle for 14 consecutive days (D1 to D17); the animals from group 2 (n = 4) received daily *p.o* administrations of Debio 1143 at 100 mg/kg for 14 consecutive days (D1 to D14) and the animals from group 3 (n = 4) received one IV injection of paclitaxel at 7.5 mg/kg every 7 days for a total of 2 injections (D7 and D14). Body weights were measured twice a week and the volumes of tumors calculated as described above. [^{18}F]-FDG-PET-CT imaging was performed in overnight fasted mice at one week of treatment (D7), two weeks of treatment (D14) and one week after treatment withdrawal (D21). Mice were anesthetized through isoflurane inhalation for intravenous injection (tail vein) of 15-20 MBq of [^{18}F]-FDG 30 minutes prior the imaging study. The mice were then maintained under anesthesia and placed on the imaging heated bed inside BioPET^{TM}-CT (Bioscan, USA). A CT scan of the same region (tumor-centered) was then obtained (150µA, 45kV, 360 projections, 8 shots/projection) and mice were maintained under anesthesia after [^{18}F]-FDG injection in order to evaluate tumor metabolism (tumor-centered, 250-700 keV). Alternatively, mice receiving vehicle, Debio 1143 or paclitaxel received an intravenous injection (tail vein) of 15-20 MBq of [^{18}F]-FDG and were immediately imaged by dynamic PET-CT for 240 seconds to evaluate tracer circulation and tumor perfusion. At the end of the last imaging, the mice were intraperitoneally injected with an overdose of pentobarbital for euthanasia and tumors harvested for gamma counting (Perkin Elmer, France). PET reconstructions were performed for the complete 30 minutes scan (late static images) and a region of interest within the tumor was manually determined and the maximum standardized uptake value (SUVmax) of the tumor measured.

Statistical analysis

All results are presented as mean +/- SEM. Statistical analysis was determined using one way ^{99m}Tc-HYNIC-Annexin V experiments) or two way ANOVA ([^{18}F]-FDG PET-CT). Analysis were performed with GraphPad Prism 6.0 (GraphPad Software Inc.), in all cases a p-value less than 0.05 was considered significant.
